# Performance and Genetic Parameters of Poplar Hybrids and Clones in a Field Trial Are Modified by Contrasting Environmental Conditions during the Vegetative Propagation Phase

**DOI:** 10.3390/plants11182401

**Published:** 2022-09-15

**Authors:** Valda Gudynaitė-Franckevičienė, Alfas Pliūra

**Affiliations:** Lithuanian Research Centre for Agriculture and Forestry, Liepų St. 1, Girionys, 53101 Kaunas, Lithuania

**Keywords:** phenolic compounds, tree growth, hybrid poplars, genetic variation, epigenetics, plant response, climate change

## Abstract

This study investigates epigenetics-like phenomena: how performance phenotypic plasticity, genotypic variation, and the heritability of growth traits and total phenolic compounds of *Populus* hybrids and clones in field trials may be modified by contrasting temperature conditions at their vegetation propagation phase. The significant effect of rooting–growing conditions on further tree performance in field trials was found for height increment in 2020, although the interaction hybrid by rooting–growing conditions was highly significant for phenolic compounds, tree height, and diameter, meaning that the performance of some hybrids was affected by rooting–growing conditions, thus demonstrating epigenetic-like effects. For phenolic compounds, interactions were also significant at the clonal level. High estimates of ecovalency indicate that some hybrids are ecologically sensitive, and epigenetic-like phenomena might occur. Hybrid *P. balsamifera* × *P. trichocarpa* is characterized by high ecovalency and specific adaptations according to mean tree height when vegetatively propagated under different rooting–growing conditions. Low estimates of *P. deltoides* × *P. trichocarpa* ecovalency demonstrate a general adaptation according to mean tree height in a field trial. Vegetative propagation conditions have also altered the genetic variation of traits in trees being planted in field trials.

## 1. Introduction

Forests are essential to our survival and well-being. Forests play a key role in mitigating climate change and its impacts on our lives. However, at the same time, climate change poses a great threat to the world’s forests—both those that already exist and those newly planted. Climate change poses new challenges to foresters, ecologists, researchers, politicians, and forest and plant sciences. Due to global warming, rising CO_2_ concentrations, and increasing precipitation in northern Europe, many deciduous tree species are expected to improve their growth rate. At the same time, other consequences of climate change, such as increased heat waves, droughts, mild winters, floods, reduced snow cover, and frozen ground depth, may be a negative factor in tree, forest, and forest ecosystem levels. These factors directly or indirectly cause stress to trees, disturb their growth rhythm and development, cause direct damages, induce defoliation, disturb physiological processes, and induce changes in the biochemical response [1,2,3,4]. When talking about trees’ adaptation to environmental conditions or survival under the influence of stressors, dendrometry parameters are often discussed, but it is very important to understand how climate affects the defensive and protective mechanisms of the plant as well. It is known that current environmental changes are much faster than climate changes in the postglacial period [5,6]. Forest trees are sessile, perennial organisms with complex life cycles often challenged by environmental variations during their long lifespan. Migration, adaptation, and phenotypic plasticity are the main strategies for tree populations surviving according to environmental changes [7,8,9]. Epigenetic phenomena also often occur here—adaptive changes due to changes in environmental conditions or stressors resulting from gene-expression changes [10,11,12]. Such severe stress can cause not only seasonal and physiological changes in trees, but through increased natural selection, the offspring’s genotype may be altered and genetic diversity reduced. Both short-term adaptation, achieved through physiological, phenotypic, and morphological plasticity, and long-term genetic adaptation are important for the survival of the plant and the entire ecosystem. Long-term genetic adaptation to large environmental changes and even species evolution can only be ensured by genetic variation and selection [13,14]. One of the biggest issues facing breeders, foresters, and biologists is the difficulty of predicting changes in tree characteristics and adaptation capacity under climate and environmental changes [15].

The significance of epigenetics in tree adaptation has been studied for some time [16,17,18]. Epigenetic processes determine alterations in gene function but do not alter the primary DNA sequence [19]. Unlike many other regulatory mechanisms, epigenetic systems have the potential to store information over time [20]. Environment impact at the embryogenesis or early development stage affects the ability of a tree to respond to not only its current environment but also to the future environment. This impact determines the manifestation of certain properties or attributes that lead the tree to survival. It is known that epigenetic changes occurring in natural populations may correlate with naturally occurring phenotypic variation [12], but this natural variation in epigenetic markers and their relationship to phenotypic traits is still not well-studied and understood. According to Bräutigam et al. [12], detailed studies and research on the epigenetics of trees and their results would help to predict the success of their adaptation to the climatic conditions of a particular area. Epigenetics is a possible way of introducing beneficial traits through plant breeding [18]. Modern forestry, new tree breeding programs, biotechnology, and even silviculture should rely on epigenetics. The ability of long-lived plants to adapt to environmental conditions in the context of global warming is crucial to both the conservation of species and ecosystems and improving their functional traits. One of the best examples of memory-controlling plant response to pathogens, herbivore attacks, or abiotic stresses is defense priming [21,22,23,24]. The priming event is followed by a period of stress memory, storing information about the priming stress through an epigenetic phenomenon and resulting in a modified response upon recurring stress exposure or a sustained response after the priming stress [23]. This memory may last several days to years for a somatic stress memory and, in some cases, may even be extended to the offspring [9]. The best-known example of epigenetic memory for forest tree species is the environmental regulation during seed production on the further performance of *Picea abies* progeny. Johnsen et al. [25] first suggested that the seed production temperature and photoperiod interact to develop a long-lasting memory mechanism regulating phenology and frost hardiness, as well as bud burst timing in *P. abies* progeny [10].

Some studies indicate that epigenetic effects can also be generated during embryogenesis or the early development of plants under vegetative propagation. According to Gömöry et al. [26], climate-induced epigenetic markings acquired during seed germination and early growth may thus be similarly durable as those acquired during embryogenesis. Furthermore, there is increasing evidence that the epigenetic state of the vegetative cell might influence the mRNA or translation profiles of the sperm [27,28]. In the review carried out by Raihan et al. [29], an important conclusion is written: all the epigenetic features (DNA methylation in nuclear and mitochondrial DNA, sRNA molecules, etc.) are associated with vegetative phase reversal. This indicates that they are synchronized to maintain epigenetic memory during vegetative propagation. The number of papers on plant epigenetics published per year is increasing, but only a small proportion of them are related to forest trees and forestry [9].

Poplar hybrids are very fast-growing trees; their wood is valuable for the paper industry and bioenergy, and it is a promising tree species both ecologically and economically [30,31]. Poplar is extremely valuable in terms of biodiversity, according to Chong et al. [32], Nilsson et al. [33], and Latva-Karjanmaa et al. [34]. *Populus* spp. plays an important role in agroforestry across Europe and North America [35]. *Populus* spp. is a worldwide tree species with a wide natural range of geographical distribution, thus growing in a variety of ecological conditions [36,37,38]. The distribution range of *P. trichocarpa* in the north and south is extremely large—it grows in America from California to Alaska. The range of *P. deltoides* in the Americas is more southern, from the Gulf of Mexico to southwestern Canada. *P. balsamifera* (Balsam poplar) is adapted to the northern climate—it grows from southeastern Canada to Alaska. Cross-breeding uses *P. maximowiczii* (Japanese poplar), native to the far east [39]. Poplar grows naturally not only in Europe and North America but also in China, India, Argentina, Chile, and Kenya [40], thus making *Populus* spp. an important tree species worldwide. Different poplars, as well as their hybrids, are successfully planted in forests, short rotation plantations, parks, along urban and rural road corridors, and in other urban territories as protective, recreational greenery worldwide [40,41,42,43]. To establish short rotation plantations or forest stands, poplars are vegetatively propagated under different environmental conditions, such as under controlled environments in greenhouses or under uncontrolled conditions outdoors in nurseries or directly in plantation areas. In greenhouses, where planting material for forests, plantations, or other greeneries is prepared, the temperature and air humidity are usually higher than on sites of the natural occurrence of poplars, and therefore transplanting to field conditions may cause stress to trees and may have an impact on their further growth and development (height, diameter), physiological, biochemical, and other processes, along with survival. To cope with environmental stresses, plants have developed different defense systems, among which the enzymatic antioxidant and non-enzymatic systems are two important forms of defense [44]. Total phenolic compounds are directly related to defense responses in the plant and are part of the non-enzymatic antioxidant system. Phenolic metabolites play an important part in other processes, for instance, incorporating attractive substances to accelerate pollination, coloring for camouflage and defense against herbivores, as well as antibacterial and antifungal activities [45,46,47]. It is known that the amount of phenolic in leaves is genetically controlled [48], but it also depends on the tree species, season, age of the leaf, environmental conditions, etc. [44,49]. It is important to find out how environmental conditions change the genetic parameters of this important defense mechanism. There is still limited information on the severity and longevity of such consequences and differences in the adaptive capacity of different hybrids and clones, given the vastly different environments in the native ranges of poplars used in breeding hybrids.

This study aimed to estimate the growth and biochemical responses of poplar hybrids and clones grown in field trials after rooting–growing under different conditions. We also sought to learn how genetic parameters of adaptive and other traits can be modified by simulated contrasting temperature conditions at their vegetative propagation phase.

## 2. Results

### 2.1. Impact of Hybrids and Treatments during Vegetative Propagation on the Total Phenolic Compound Content in Hybrid Populus Leaves in a Clonal Field Trial

An analysis of variance showed that there was a highly significant impact of growing conditions during the vegetative propagation phase in trees planted in the field trial (*p* < 0.001) on the total phenolic compounds (Table 1).

**Figure 1:**
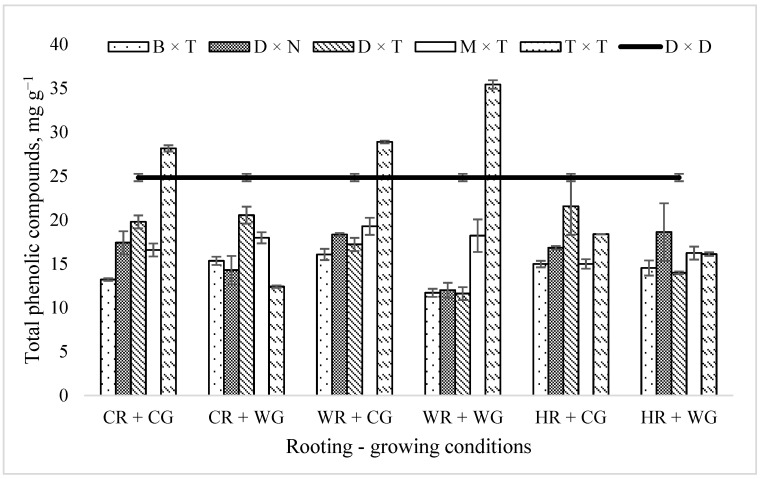
Total phenolic compounds amount (mg g^−1^) in *Populus* hybrids in field trial vegetatively propagated under different rooting–growing conditions. Rooting–growing condition abbreviations: cool rooting and cool growing conditions (CR + CG), cool rooting and warm growing conditions (CR + WG), warm rooting and cool growing conditions (WR + CG), warm rooting and warm growing conditions (WR + WG), hot rooting and cool growing conditions (HR + CG), and hot rooting and warm growing conditions (HR + WG). Hybrid-type abbreviations: B × T—*P. balsamifera* × *P. trichocarpa*, D × N—*P. deltoides* × *P. nigra*, D × T—*P. deltoides* × *P. trichocarpa*, M × T—*P. maximowiczii* × *P. trichocarpa*, T × T—*P. trichocarpa* × *P. trichocarpa*, D × D—*P. tremula*.

There was also a very significant impact of rooting × growing conditions, rooting conditions × clone, growing conditions × clone, and rooting × growing conditions × clone interactions on the total phenolic compounds. However, the effect of plant rooting conditions of cuttings in the Phytotron greenhouse during vegetative propagation on the amount of total phenolic compounds in trees planted in the field trial was not significant (*p* < 0.1003) (Table 1). Clone interactions with the different treatments indicate differences in the genetic response of clones to changes in environmental factors after transplanting to field trials. The maximum amount of total phenolic compounds in the field trial was observed when vegetatively propagated under WR + CG (18.96 ± 0.77 mg g^−1^) and CR + CG (18.48 ± 0.89 mg g^−1^) conditions, while the lowest amount was under WR + WG (15.44 ± 1.39 mg g^−1^) (Figure 1).

Analysis of variance (ANOVA) showed that hybrid and hybrid × rooting–growing conditions interaction had a highly significant impact on total phenolic compounds (*p* < 0.001) (Table 2). On the other hand, the impact of treatment during vegetative propagation (rooting + growing conditions) in the Phytotron greenhouse had an insignificant impact on total phenolic compounds (Table 2). 

The largest differences in the amount of phenolic compounds between hybrids were observed under WR + WG conditions. Under HR + WG conditions, there are minimal differences between the hybrids in the amount of total phenolic compounds. 

As shown in estimates of hybrids’ ecovalency, the largest impact of rooting and growing treatments in the greenhouse under the vegetative propagation stage on the amount of phenolic compounds transplanted to field trial trees was observed for hybrid *P. trichocarpa* × *P. trichocarpa* (*ew* = 314.8). The lowest impact of rooting and growing treatments in the greenhouse under the vegetative propagation stage was in *P. deltoides* × *P. nigra* (*ew* = 14.2) (Table 3).

The highest mean total phenolic compound quantity (23.23 mg g^−1^) was obtained for the *P. trichocarpa* × *P. trichocarpa* hybrid. This hybrid had the highest total phenolic compounds content in the field trials when propagated under WR + WG, while it had the lowest under CR + WG conditions (Figure 1). WR + WG conditions did not cause such stress for other hybrids. The lowest level of mean total phenolic compounds was obtained in the *P. balsamifera* × *P. trichocarpa* hybrid. Under WR + WG conditions, it was 11.7 ± 0.46 mg g^−1^ (Figure 1). A lower (11.61 ± 0.73 mg g^−1)^ amount of total phenolic compounds was only obtained in the *P. deltoides* × *P. trichocarpa* hybrid under the same WR + WG conditions. WR + WG conditions resulted in the lowest total phenolic compounds level among most hybrids. 

In aspen, as a control tree, total phenolic compounds reached 24.84 ± 0.42 mg g^−1^. Only the *P. trichocarpa* × *P. trichocarpa* hybrid under WR + WG, WR + CG, and CR + CG conditions had a higher level of total phenolic compounds, 35.43 ± 0.48, 28.89 ± 0.15, and 28.16 ± 0.36 mg g^−1^, respectively (Figure 1).

### 2.2. Dependence of the Growth Rate of Poplar Hybrids in a Clonal Field Trial on the Vegetative Propagation (Rooting–Growing) Conditions

Analysis of variance (ANOVA) showed that the effect of treatment during vegetative propagation (rooting + growing conditions) in the Phytotron greenhouse and the effect of the hybrid on tree diameter and height increment in planted trees in the 2021 field trial was significant (0.01 < *p* < 0.05, Table 2). Hybrid × rooting–growing conditions interaction had a significant impact on tree diameter (*p* < 0.001), height (0.001 < *p* < 0.01), and height increment in 2020 (0.001 < *p* < 0.01), but not on height increment in 2021. The impact of treatments of cuttings in the greenhouse had a highly significant impact on height increment in 2020 (*p* < 0.001) but did not have a significant impact on tree height, diameter, and height increment in 2021 (Table 2). The hybrid × rooting–growing conditions interaction remained significant in terms of diameter.

**Figure 2:**
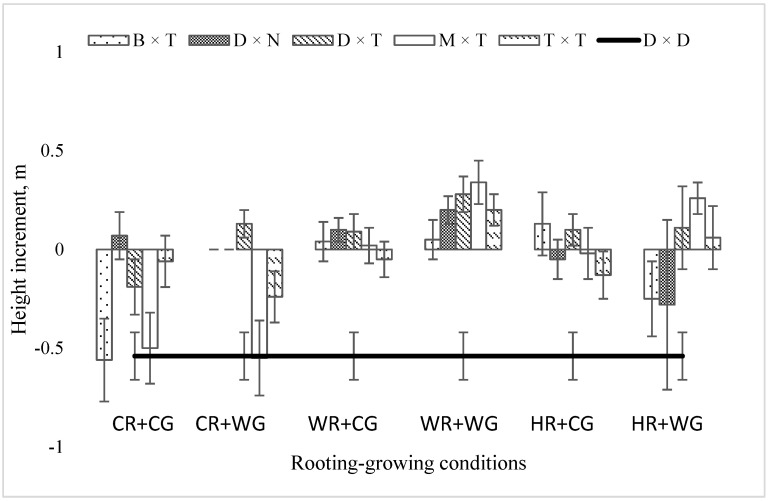
Mean height increment (m) of *Populus* hybrids in field trial in 2020, which were vegetatively propagated under different rooting–growing conditions. Rooting–growing condition abbreviations: cool rooting and cool growing conditions (CR + CG), cool rooting and warm growing conditions (CR + WG), warm rooting and cool growing conditions (WR + CG), warm rooting and warm growing conditions (WR + WG), hot rooting and cool growing conditions (HR + CG), and hot rooting and warm growing conditions (HR + WG). Hybrid-type abbreviations: B × T—*P. balsamifera* × *P. trichocarpa*, D × N—*P. deltoides* × *P. nigra*, D × T—*P. deltoides* × *P. trichocarpa*, M × T—*P. maximowiczii* × *P. trichocarpa*, T × T—*P. trichocarpa* × *P. trichocarpa*, D × D—*P. tremula*.

In 2020 (after two vegetation seasons), trees in the field still experienced growth disturbances caused by planting stress, and only in 2021 were there positive changes in growth. In 2020, the mean height increment along all the hybrids and conditions was not positive. Since many trees suffered and were in poor condition—withered or bitten tops, damaged bark, dried up—their mean height was smaller than at planting time. The greatest mean height increment in 2020 was for trees vegetatively propagated under warm rooting and warm growing (WR + WG) conditions—21 cm (Figure 2). All the hybrids kept growing under WR + WG conditions, except the control tree—aspen. The greatest losses in mean height increment after planting in the field were in hybrids vegetatively propagated under CR + CG conditions. Only the *P. deltoides* × *P. nigra* mean height increment was positive (4 cm). The greatest mean height increment in 2020 was obtained in the *P. deltoides* × *P. trichocarpa* hybrid—it reached 8 cm per season (Figure 2). This hybrid grew well in the field when propagated under warm rooting and warm growing (WR + WG) conditions but suffered great height losses when propagated under cold rooting and cold growing (CR + CG) conditions. The *P. balsamifera* × *P. trichocarpa* hybrid experienced the greatest growth stress in 2020.

**Figure 3:**
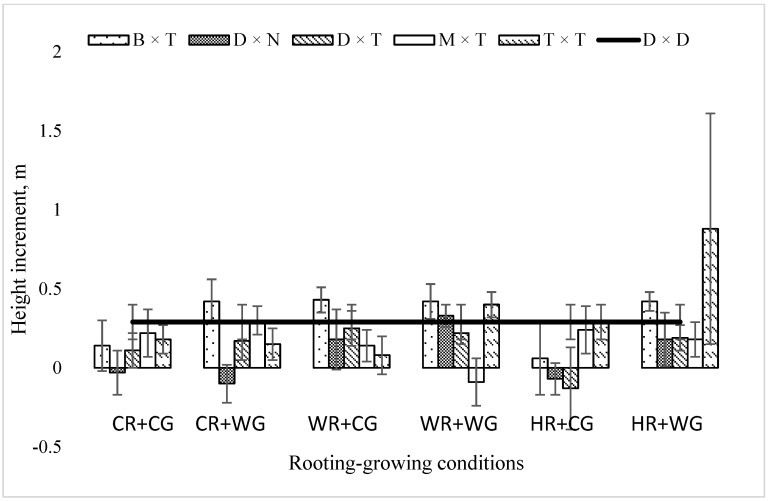
Mean height increment (m) of *Populus* hybrids in field trial in 2021, which were vegetatively propagated under different rooting–growing conditions. Rooting–growing condition abbreviations: cool rooting and cool growing conditions (CR + CG), cool rooting and warm growing conditions (CR + WG), warm rooting and cool growing conditions (WR + CG), warm rooting and warm growing conditions (WR + WG), hot rooting and cool growing conditions (HR + CG), and hot rooting and warm growing conditions (HR + WG). Hybrid type abbreviations: B × T—*P. balsamifera* × *P. trichocarpa*, D × N—*P. deltoides* × *P. nigra*, D × T—*P. deltoides* × *P. trichocarpa*, M × T—*P. maximowiczii* × *P. trichocarpa*, T × T—*P. trichocarpa* × *P. trichocarpa*, D × D—*P. tremula*.

After three vegetation seasons in the field, the greatest mean height increment was obtained for hybrids vegetatively propagated under heat rooting and warm growing (HR + WG) and warm rooting and warm growing (WR + WG) conditions, 37 cm and 25 cm, respectively (Figure 3).

All the hybrids propagated under these two conditions grew well in the field and demonstrated a positive mean height increment after the third vegetation season. The lowest mean height increment in the field trial was obtained for hybrids vegetatively propagated under heat rooting and cold growing (HR + CG) conditions (Figure 3). The greatest mean height increment was reached in *P. trichocarpa* × *P. trichocarpa*—88 cm, while the lowest (18 cm) was reached in *P. maximowiczii* × *P. trichocarpa* and *P. deltoides* × *P. nigra* (Figure 3). As a control tree, aspen had a mean height increment of 29 cm, which was higher than the mean height increment of the entire experiment. 

**Figure 4:**
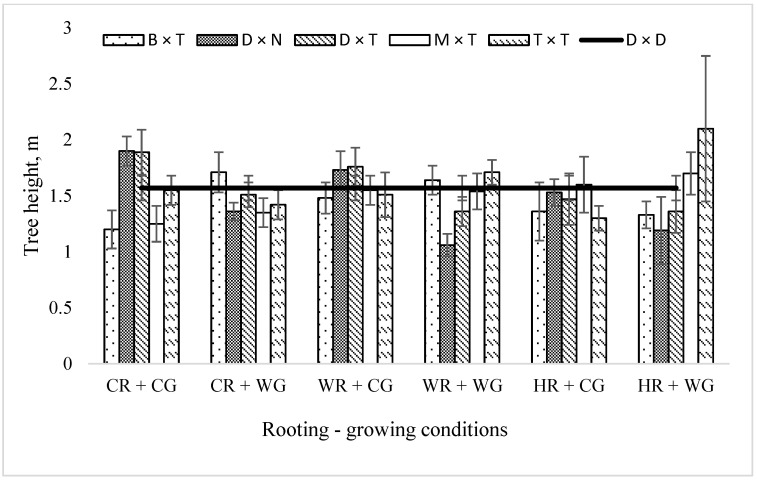
Mean height (m) of *Populus* hybrids in field trial in 2021, which were vegetatively propagated under different rooting–growing conditions. Rooting–growing condition abbreviations: cool rooting and cool growing conditions (CR + CG), cool rooting and warm growing conditions (CR + WG), warm rooting and cool growing conditions (WR + CG), warm rooting and warm growing conditions (WR + WG), hot rooting and cool growing conditions (HR + CG), and hot rooting and warm growing conditions (HR + WG). Hybrid type abbreviations: B × T—*P. balsamifera* × *P. trichocarpa*, D × N—*P. deltoides* × *P. nigra*, D × T—*P. deltoides* × *P. trichocarpa*, M × T—*P. maximowiczii* × *P. trichocarpa*, T × T—*P. trichocarpa* × *P. trichocarpa*, D × D—*P. tremula*.

The highest mean tree height was obtained for trees vegetatively propagated under warm rooting and cool growing (WR + CG) and cool rooting and cool growing (CR + CG) conditions, 1.60 and 1.55 m, respectively, and the lowest mean tree height was obtained for trees propagated under heat rooting and cold growing (HR + CG) conditions—1.45 m (Figure 4). The tallest hybrid among all the trees was *P. trichocarpa* × *P. trichocarpa*. The mean height among all conditions reached 1.59 m, which exceeded the average height of aspen by 157 cm (as a control tree species).

In our study, the mean height of hybrid poplars propagated under HR + CG conditions, even after three vegetation seasons of planting in the field trial, was the lowest, as well as the mean height increment in 2021.

The mean height increment of *P. deltoides* × *P. trichocarpa* in 2020 was 8 cm (greatest among all hybrids); in 2021, it was 19 cm (one of the lowest). The mean height of the hybrid was 2 cm lower than the trial mean but still one of the greatest among all hybrids. The *P. deltoides* × *P. trichocarpa* hybrid was characterized by the best survival among all hybrids after three growing seasons.

As shown in estimates of the hybrids’ ecovalency, the largest impact of rooting and growing treatments in the greenhouse in the vegetative propagation stage on height growth in trees transplanted to the field was observed for the *P. balsamifera* × *P. trichocarpa* (*ew* = 0.6) and the *P. deltoides* × *P. nigra* (*ew* = 0.4) hybrids, while the lowest impact was observed in the *P. deltoides* × *P. trichocarpa* hybrid (*ew* = 0.2, Table 3).

*The P. trichocarpa* × *P. trichocarpa* hybrid is characterized by the greatest mean height. The best result was obtained when propagated under HR + WG conditions—the mean height reached 2.1 m. *P. trichocarpa* × *P. trichocarpa* vegetatively propagated under HR + CG demonstrated the lowest mean height among all propagation environments (Figure 4), which may indicate the stress experienced by the hybrid under sudden changes in temperature.

### 2.3. Changes in Genetic Parameters of Growth Traits and Total Phenolic Compounds of Trees in the Clonal Field Trial Depending on Rooting–Growing Treatments during Vegetative Propagation in the Phytotron Greenhouse

The clonal component of variation, which shows the share of clonal genetic variation in the overall variability of traits, ranged from 94.04 to 99.55% in terms of the total phenolic compounds under different treatments (rooting–growing conditions) (Table 4). The highest clonal variation component—99.55%—was obtained under WR + WG conditions, while the lowest was under CR + WG conditions. There were no highly pronounced differences between clonal components in variation under different environments. The lowest genotypic variation was found under WR + CG conditions (*CVg* = 22%, Table 5). The highest genotypic variation in the field trial was found under WR + WG − *CVg* reached 51.3% (Table 5).

The highest heritability coefficient was obtained for total phenolic compounds under WR + WG conditions (*H_i_*^2^ = 1.00), and the lowest was obtained for that under CR + CG treatment (*H_i_*^2^ = 0.95) (Table 5).

The coefficient of genotypic variation of traits ranged from 0% to 227.3% for different traits under different treatments (rooting–growing conditions) (Table 5). The highest coefficient of genotypic variation, 227.3%, was obtained for height increment under HR + CG conditions, while the lowest, 0%, was obtained for height increment under WR + CG conditions. All traits under WR + CG conditions were characterized by the lowest coefficient of genotypic variation among all treatments. The highest individual heritability coefficient was obtained for a diameter under WR + WG conditions (*H_i_*^2^ = 0.42), and the lowest was obtained for a height increment (*H_i_*^2^ = 0.00) and diameter (*H_i_*^2^ = 0.01) under WR + CG treatment (Table 5). The greatest phenotypic variation was obtained for height increments, while the lowest was obtained for height.

## 3. Discussion

The amount of total phenolic compounds is tightly controlled genetically; however, growing conditions and their interaction may determine changes in the concentration of total phenolic compounds. Environmental stresses, such as high light or UV radiation, low temperatures, pathogen infection, herbivores, heavy metals, nutrient deficiency, and increased production of free radicals and other oxidative species in plants, lead to phenolic concentration changes in plants [50]. In our study, we obtained different amounts of total phenolic compounds in field trials when vegetatively propagated under different conditions. Clone interactions with the different treatments indicate differences in the genetic response of clones to changes in environmental factors after transplantation to field trials.

In our study, the highest mean total phenolic compound quantity (23.23 mg g^−1^) was obtained for the *P. trichocarpa* × *P. trichocarpa* hybrid. As shown in estimates of the hybrids’ ecovalency, the greatest impact of rooting and growing treatments in the greenhouse under the vegetative propagation stage on the amount of phenolic compounds in trees transplanted to the field trial was observed for the *P. trichocarpa* × *P. trichocarpa* hybrid (*ew* = 314.8). This indicates that this hybrid has the highest ecological sensitivity. Although the natural *P. trichocarpa* range extends to California, it is most prevalent in cooler climate zones and the mountains and reaches Alaska [51]; therefore, warm propagation and growing conditions may have unbalanced biochemical processes. The genotype determines the amount of phenolic compounds not only in hybrid poplars, as we found in our study, but also in raspberries [52], pears [53], and oaks [54].

Phenolic compounds are secondary metabolites, and they play a significant protective–defensive role in plants. Metabolic changes play vital roles in plant acclimatization and adjustment to temperature stresses. They preserve leaf physiological processes during high-temperature stress [55]. Hale et al. [56] observed an increased concentration of phenolic glycosides in response to drought stress in *Populus*. In *Populus* spp., biotic stress has commonly been associated with levels and salicinoid phenolic glucosides (SPGs) [57,58], and these compounds are often related to environmental stress responses and performance [59,60]. In our study, *P. trichocarpa* did not experience biotic stress, but abiotic stress indicates an increased risk of pests, diseases, herbivores, etc., and the plant intensified the production of phenolic compounds.

The lowest level of mean total phenolic compounds in our study was obtained in the *P. balsamifera* × *P. trichocarpa* hybrid. It is known that the *P. balsamifera* × *P. trichocarpa* hybrid is characterized by a high plasticity level [4]; this shows that trees can respond to and adapt to climate and environmental change in a relatively short time without suffering. Furthermore, the stable and low amount of phenolic compounds shows that rooting–growing conditions did not cause long-term stress to the hybrid and did not intensify the defense mechanism.

A lower amount of total phenolic compounds was only obtained in the *P. deltoides* × *P. trichocarpa* hybrid under the same WR + WG conditions. WR + WG conditions resulted in the lowest total phenolic compounds level among most hybrids. This can be explained by the fact that both rooting and growing conditions were the same (warm), and the plants did not experience a sudden temperature change. In Sobuj et al.’s [61] study, elevated temperature reduced the concentration of total phenolic in the stem bark of *P. tremula* since warming stimulated the growth of the aspen.

Adverse environmental conditions or sudden changes in the environment unbalance biochemical processes. The reaction of the plant depends on clones and their different phenotypic plasticities. Non-optimal conditions can reduce the genetic variation of many traits, which is important in the adaptation process. The greater the genetic variation of adaptive traits, the greater the potential for genetic adaptation [62]. Although the content of phenolic compounds in plants is not a primary indicator of adaptation, they are directly related to the protective mechanism of the plant [63]. Plants have developed the ability to produce an enormous number of phenolic secondary metabolites, which are not required in the primary processes of growth and development but are vital for their interaction with the environment, reproductive strategy, and defense mechanisms [48].

In our study, the mean height of hybrid poplars propagated under HR + CG conditions, even three vegetations seasons after planting in the field trial, was the lowest, as well as the mean height increment in 2021. This shows that stress (huge temperature differences in a short period) experienced during vegetative propagation also affects the further growth rate of the tree. Increased soil temperature often causes stress and is a limiting factor for growth: soil macro- and microorganisms die because of the unfavorable temperature of the soil [64], and heat increases root hydraulic conductivity up to a level harmful to plant functions [65], etc. Therefore, a tree’s ability to grow under stressful conditions is important for adaptation. Raj et al. [66] found that there is a nursery effect on stress response in a common controlled environment for three economically important poplar hybrids *(P. deltoides* × *P. nigra*, *P. deltoides* var. occidentalis × *P. laurifolia* × *P. nigra*, and *P. laurifolia* × *P. nigra*) genotypes. The genotypes each have a distinct propagation history that has led to different paths of adaptation and growth. Our findings support Raj et al.’s [66] studies and the hypothesis that the stress response of a given poplar genotype can be shaped by the history of that clone and epigenetics. 

Some hybrids, such as *P. deltoides* × *P. trichocarpa*, can keep their growth rhythm stable according to changing conditions. The mean height increment of *P. deltoides* × *P. trichocarpa* in 2020 was 8 cm (greatest among all hybrids), and in 2021, it was 19 cm (one of the lowest). This hybrid was characterized by the best survival among all hybrids after three growing seasons. *P. deltoides* × *P. trichocarpa* is not very sensitive to environmental conditions at any level. This is confirmed by the lowest impact of rooting and growing treatments in the greenhouse during the vegetative propagation stage on height growth in trees transplanted to the field trial among all the hybrids (*ew* = 0.2). In our previous studies [67], *P. deltoides* × *P. trichocarpa* demonstrated the lowest ecovalence in height among 10 different poplar hybrids as well. Ecovalence characterizes the relative lability of a hybrid in relation to other hybrids and describes what part of the G × E interaction is determined by the ecogenetic variability of one or another hybrid and shows how strongly the genotype response varies to different trial conditions. High ecovalence shows growth losses when environmental conditions are unfavorable, but under favorable conditions, growth can be good.

In our study, the *P. trichocarpa* × *P. trichocarpa* hybrid is characterized by the greatest mean height. Even though *P. trichocarpa* is mostly of northern origin, the best result was obtained when it was propagated under HR + WG conditions. This could be explained by epigenetics phenomena—after hot/warm conditions during propagation, *P. trichocarpa* × *P. trichocarpa* bud set earlier. Liu & El-Kassaby’s [68] study shows evidence that *P. trichocarpa* can increase fitness via an increase in the active growth rate (biomass) and is likely to extend its bud set and entire growth period as responses to less-limiting temperatures (including less-frequent frost events) due to climate change, reconciled by abbreviating the duration from the final bud set to the onset of leaf drop and increased drought resistance via an increase in water-use efficiency. *P. trichocarpa* × *P. trichocarpa* vegetatively propagated under HR + CG demonstrated the lowest mean height among all propagation environments, which may indicate the stress experienced by the hybrid under sudden changes in temperature. Temperature, such as the photoperiod, regulates plant phenology and growth. Sudden changes in temperature or extreme temperatures unbalance the growing rhythm. According to Apuli et al. [69], incorrect timing of phenology transitions results in a loss of potential growth through extended dormancy or loss of realized growth in the form of damage to important tissues, such as meristems and leaves from exposure to unfavorable conditions, or even death. Dormancy hence represents an important life history tradeoff between growth and survival. Maladapted individuals are likely to suffer lowered reproductive success and/or biomass production, both of which may have large ecological and economic repercussions [70]. High mean values of the heritability coefficient indicate pronounced genetic differences between clones, strong genotypic control of the trait, and less of an impact suffered by random factors. In our study, we obtained a very high heritability coefficient for the total phenolic amount. Other authors have obtained similar results. A lower heritability coefficient in *P. tremula* at the population level was obtained by Robinson et al. [71]. The heritability coefficient of the phenolic amount depends not only on the environmental conditions but also on the season and tree conditions [72]. Stevens and Lindroth [72] obtained high values of the heritability coefficient for phenolic compounds, which decreased in defoliated trees twice and was lower in August compared to July. 

All genetic parameters (except the heritability coefficient) in the field trial changed depending on the treatment at the vegetative propagation phase, and this might be due to stress memory or epigenetics. The role of epigenetics in phenolic-related processes is still not sufficiently researched. The very recent report provides mechanistic evidence of the epigenetic regulation of flavonoid biosynthesis under UV-B radiation in *A. annua* L. [73]. It was found that epigenetics plays a role in anthocyanin biosynthesis in potato cell culture [74].

Our study shows that vegetative propagation conditions alter the genetic variation of traits in trees planted in a field trial. High genetic variation makes it easier for a species to adapt to environmental conditions [75] and is one of the guarantors of vegetation sustainability, along with high genetic diversity; many different gene variants can recombine into genotypes that are suitable for an ever-changing environment during sexual reproduction, thus guaranteeing the species’ adaptation and survival [76,77]. In our studies, WR + CG conditions resulted in a decrease in genetic variation in very important traits—height and height increment. Differences in genotypic variation in different environmental conditions are determined by an uneven biochemical process disruption rate of different clones and their different phenotypic plasticities. Genetic diversity is decreasing due to habitat degradation and population loss, unsustainable harvest, invasive species, and increasing extreme climatic events, which is a worldwide problem [78]. 

In our study, we obtained a low heritability coefficient for height, height increment, and diameter. Heritability is known to vary between different environments, though, for most species and traits [79]. It is known that drought and frosts also unbalance the heritability of the height, diameter, and survival of *Populus* hybrids [4]; the heritability of the amount of dry biomass depends on site conditions [80], while the heritability of volume and diameter in breast height depends on latitude [81] in *Populus*. The low values of heritability coefficients show that in these environmental conditions (after different treatments during propagation), the random ecological variation of traits was higher than the genotypic variation. The weak heritability coefficient indicates a strong interaction between the clone and the environment as well. This suggests that clones differ in their ecogenetic response to changes in ecological conditions, i.e., they are characterized by a specific ecogenetic plasticity. Strong interaction between the clone and the environment can lead to growth and adaptivity decreases in future progenies [82].

In our study, we obtained a high phenotypic variation for height increment. This indicates that this trait is determined not only by genetic factors but also by ecological factors. It is highly dependent on the heterogeneity of environmental conditions in the field trial or, in our case, rooting–growing conditions in the Phytotron greenhouse. It is known that coordinated genetic–epigenetic adaptive differentiation influences primary phenotypic diversity during epigenetic processes in adaptation and evolution [12]. Phenotypic variation of all traits varied with environmental conditions in our study. The phenotypic variation in field trials for tree diameter was more significantly influenced by growing conditions, and for height, it was more significantly influenced by rooting conditions. These changes in phenotypic variation could be determined by epigenetics. There are reports indicating that epigenetic change can cause phenotypic variation, and thus epigenetic change can be considered an important factor in understanding phenotypic change [83]. Heritable genetic variation in plant traits represents the raw material for future adaptive evolution. The contribution of heritable genetic variation to total phenotypic variation is essential for evolutionary ecology. Its importance rises even more under global climate change and stressful environmental conditions, as it is unclear to what degree terrestrial plant species can adapt to different habitat qualities [84].

We can observe that in 2021, the effect of the interaction of hybrid × rooting–growing conditions on height increment decreased compared to 2020. This shows that the impact of epigenetic phenomena on some hybrids decreased, and the impact of hybrids became higher. For the diameter, hybrid × rooting–growing conditions interaction remains significant, proving that propagation conditions have differing effects on different hybrids’ productivity (biomass, volume). Epigenetic phenomena can change over time. It is a dynamic process; therefore, traits may “wash out” over several generations [85], and at the same time, the epigenetic changes in plants can be inherited over generations in the form of epialleles [86]. According to Latzel et al. [87], plants can predict future conditions based on their past experiences. Plants fix and pass epigenetic changes from generation to generation, which are stored in the cell memories [87]. This means that the rooting–growing conditions help the plant accumulate experience; later, if the same conditions are repeated, they will help it adapt and change its phenotype via epigenetic phenomena.

## 4. Materials and Methods

### 4.1. Plant Material

This study was performed on 20 cultivars and experimental clones of intraspecific crosses of poplars (*P. trichocarpa* (Torr. & Gray.) and 4 different interspecific hybrids of poplars (*P. deltoides* L. × *P. nigra*, *P. deltoides* × *P. trichocarpa*, *P. maximowiczii* A. Henry × *P. trichocarpa*, and *P. balsamifera* L. × *P. trichocarpa*) with distinguished bioecological characteristics (Table 6). The clones were selected from the clonal collection of hybrid poplars at the LAMMC Institute of Forestry, Kaunas district, central Lithuania. Clones were vegetatively propagated in the Phytotron of LAMMC Institute of Forestry. Aspen (*P. tremula* L.) was planted in the field trial as a native control tree species.

### 4.2. Design of Experiment

Hybrid poplar clones for the testing in the clonal field trial were vegetatively propagated by rooting of cuttings under different environmental conditions (treatments) set in an automated Phytotron greenhouse. Cuttings (15–17 cm in length) of each clone were planted into the squared plastic pots (15 × 15 × 20 cm) filled with 3.5 l of peat soil (Klasmann KTS-1) that were placed on irrigation tables. One-third of ramets were rooted in pots outdoors under natural conditions, one-third in the Phytotron greenhouse, and one-third in the Phytotron greenhouse with the additional electric heating of pots with substrate from below. During the cutting rooting phase of vegetative propagation, the average air temperature in the greenhouse was 25 °C; outdoors, it was 19 °C. The average soil temperature outdoors was 19 °C; in the greenhouse, 22 °C; and in pots with additional heating, 24 °C. Air humidity was kept between 65 and 85% using an automated fog sprinkle system. The plants were regularly watered from below by temporary (0.5 h a day) flooding pots on irrigation tables to fully saturate the soil and keep the soil moisture at 80–95% of the full moisture capacity (FMC) throughout the experiment. In the middle of the growing season, the growing conditions were changed: half of the ramets that sprouted in the greenhouse were moved to grow outdoors, half of the ramets that were rooted outdoors were moved to the greenhouse, and heating of roots was turned off while they continued to grow in the greenhouse. The rest of them were moved outdoors. This resulted in six temperature treatments/regimes during vegetative propagation: cool rooting and cool growing conditions (CR + CG), cool rooting and warm growing conditions (CR + WG), warm rooting and cool growing conditions (WR + CG), warm rooting and warm growing conditions (WR + WG), hot rooting and cool growing conditions (HR + CG), and hot rooting and warm growing conditions (HR + WG). 

### 4.3. Measurements and Total Phenolic Compounds Extraction

The next spring (in 2019), the trees were planted in a clonal field trial in the Jonava forest district of the State Forest Enterprise in Jonava district, central Lithuania. The location is in the lowlands of central Lithuania. The average annual rainfall is 572 mm, and the mean temperature is 6.5 °C. A clonal trial was established in a randomized complete block design. Clones were planted in row plots containing 5 to 10 trees. Trees were planted with 2.6 m spacing between rows and 2 m within rows. In total, over 1000 trees were planted. Each clone was represented by 60–70 plants. Tree height and stem diameter at the root collar were measured at the beginning of the growing season in 2019 and the end of growing seasons in 2019, 2020, and 2021. The total content (concentration) of phenolic compounds (mg g^−1^) in microgreen fresh matter was determined by preparing methanolic extracts (fresh tissue ground with liquid nitrogen and diluted in 80% methanol at the ratio 1:10 (m/v)) and using a colorimetric Folin–Ciocalteu method [88]. Absorbance was measured at 765 nm using a Genesys 6 spectrophotometer (Thermospectronic, Waltham, MA, USA) against water as a blank. Total phenolic contents were determined by a calibration method using gallic acid as a standard.

### 4.4. Statistical Analysis

To estimate the significance of the effects of various factors—treatments (rooting conditions and growing conditions), blocks, clones, and hybrids and their interaction with treatments— multifactor variance analysis was performed on single-tree data using the MIXED procedure (procedure option—“Covparms“) in SAS v.9.4 software [89], which is based on mixed-model equations (MME) and the restricted maximum likelihood (REML) method. The following linear models were used for the joint analyses (1,2) of the treatments and the separate analyses (3) of an individual treatment:

*y_jklmn_* = *µ* + *tr_j_* + *tg_k_* + *tr_j_* × *tg_k_* + *h_n_* + *h_n_* × *tr_j_* + *h_n_* × *tg_k_* + *b_m_* + *ε_jklmn_*, (1)

*y_lnjk_* = *µ* + *h_n_* + *tr_jk_* + *h_n_* × *tr_jk_* + *b_m_* + *ε_ljnk_*, (2)

*y_im_* = *µ* + *c_i_* + *b_m_* + *ε_im_*, (3)

where *y_jklmn_* is an observation on the *l*th ramet from the *n*th hybrid in the *m*th block in the *j*th rooting and *k*th growing treatment; *y_lnjk_* is an observation on the *l*th ramet from the *n*th hybrid in the *jk*th treatment in the *m*th block; *y_ilm_* is an observation on the *i*th ramet from the *i*th clone in the *m*th block; *μ* is the overall mean; *tr_j_* is the fixed effect due to the *j*th rooting treatment; *tg_k_* is the fixed effect due to the *k*th growing treatment; *b_m_* is the fixed effect due to the *m*th block; *tr_j_ × tg_k_* is the fixed effect of *j*th rooting × *k*th growing treatments interaction; *h_n_* is the fixed effect due to the *n*th hybrid; *h_n_ × tr_jk_* is the fixed effect due to the *n*th hybrid × *jk*th treatment interaction; *h_n_ × tr_j_* is the fixed effect of the interaction of the *n*th hybrid × *j*th rooting treatment; *h_n_ × tg_k_* is the fixed effect of the interaction of the *n*th hybrid × *k*th growing treatment; *c_i_* is the random effect due to the *i*th clone; and *ε_ijklm_, ε_ljnk_,* and *ε_iklm_* are the random residuals. The model assumes that the random effects are normally distributed with the expectation of zero and corresponding variances: $\sigma_{c}^{2}$, $\sigma_{c*tr}^{2}$, $\sigma_{c*tg}^{2}$, $\sigma_{c*b}^{2}$, and $\sigma_{e}^{2}$*σ*. Assumptions of normal distribution of residuals and variance homogeneity were tested using the GLM and UNIVARIATE procedures in SAS software (SAS Institute, 2020). Statistical significance of the effects of fixed factors—treatments, blocks, and interactions between treatments and blocks—was estimated by a P-test using the MIXED procedure in SAS software [89]. Z-tests were used to determine where random effects were significantly different from zero. Least-squares means estimates were obtained for treatments, as well as for hybrids and clones in each treatment. Statistical significance (at *p* < 0.05) of differences between least-squares means was tested using a *t*-test and the MIXED procedure in SAS software [89].

Using statistical model 2, clonal variance components were estimated as:

(4) $$VC_{c}^{2}=\sigma_{c}^{2}/\left(\sigma_{c}^{2}+\sigma_{e}^{2}\right),\;$$

where $VC_{c}^{2}$ is the clonal variance component, $\sigma_{c}^{2}$ is the clonal variance, and $\sigma_{e}^{2}$ is random residual. The variance component of each effect was expressed as a percentage of the dispersion of all analyzed random effects (included in the model). Genetic parameters were estimated using the results of variance analysis separately for each treatment. The clonal heritability coefficient on the level of individuals for each trait was calculated by the following formula:

(5) $$H_{i}^{2}=\sigma_{ci}^{2}/\sigma_{phen\;}^{2}$$

where $H_{i}^{2}$ is the coefficient of individual clonal heritability, $\sigma_{i}^{2}$ is the clonal variance, and $\sigma_{i}^{2}$ is the phenotypic variance. The standard errors of the heritability coefficient under an unequal number of trees per family were calculated based on Becker (1984). The clonal heritability coefficient (repeatability) on the level of means was estimated using the following formula:

(6) $$H_{m}^{2}=\sigma_{c}^{2}/(\sigma_{c}^{2}+(\sigma_{e}^{2}/k))$$

where $H_{m}^{2}$ is the clonal heritability coefficient on the level of means, $\sigma_{c}^{2}$ is the clonal variance, $\sigma_{e}^{2}$ is the random variance, and *k* is the coefficient showing the mean number of trees per clone. The errors of heritability coefficients were estimated according to Swiger et al.’s [90] method modified by Becker [91] for an uneven number of observations. The genotypic variation coefficient in every clonal trial was estimated based on Falconer et al. [92] and Falconer [93].

To evaluate the stability of individual hybrids across Phytotron treatments and the contribution of each of the hybrids to the hybrid × treatment interaction variances, the Wricke ecovalence values [94] were calculated using hybrids’ least-squares means obtained within each site, using the “lsmeans” option of the SAS MIXED procedure. The ecovalence value for each hybrid was expressed as a percent of the total hybrid × treatment interaction variance. This analysis was conducted for traits where hybrid × treatment interaction was significant. The Shukla stability variances were computed, and the statistical significance of the ecovalences was tested using the F-test developed by Shukla [95]. In calculating ecovalences to better fulfill the assumptions behind the linear model and thus reduce the scale effects of different sites in a joint analysis, the data were transformed to equal genotypic variance using the method of Danell [96]. For each treatment, the assessed values for each tree were multiplied by a scaling factor, which for the *i*th treatment, was ¼ sC = sci, where sC and sci are the mean clonal genotypic standard deviations over both treatments and for the *i*th treatment, respectively. The phenotypic plasticity of each hybrid was estimated as the difference between the maximum and minimum least-squares means obtained within each treatment.

## 5. Conclusions

A significant effect of rooting–growing conditions at the vegetative propagation phase on further tree performance in the field trial was found for a height increment in 2020, although the interaction hybrid of rooting–growing conditions was highly significant for phenolic compounds, tree height, and diameter, meaning that the performance of some hybrids was modified by rooting–growing conditions, thus demonstrating epigenetic-like effects. For phenolic compounds, interactions were also significant at the clonal level.

The largest impact of rooting and growing treatments in the Phytotron greenhouse at the vegetative propagation stage on the amount of phenolic compounds in the trial of trees transplanted to the field was observed for the *P. trichocarpa* × *P. trichocarpa* hybrid–total phenolic amount reached 23.23 mg g^−1^. High estimates of hybrids’ ecovalency reaching up to 314.8 for phenolic compounds indicate that this hybrid is ecologically sensitive, and epigenetic-like phenomena may occur here.

The *P. balsamifera* × *P.trichocarpa* hybrid is characterized by high tree height ecovalency and specific adaptation resulting from different rooting–growing conditions of vegetative propagation. Low estimates of *P. deltoides* × *P. trichocarpa* ecovalency demonstrate a general type of adaptation.

After three vegetation seasons in the field, the greatest mean height increments reaching 37 cm and 25 cm were obtained for vegetatively propagated hybrids under heat rooting and warm growing and warm rooting and warm growing conditions, respectively.

Different vegetative propagation conditions altered the genetic variation of traits in trees planted in the field trial. The genetic variation in height increment was strongly unbalanced from 0.0 under WR + CG conditions till 227.3 under HR + CG. The heritability of the main growth traits (height, height increment, and diameter) was extremely unbalanced due to different rooting–growing conditions. Furthermore, the heritability of total phenolic compounds was impacted by rooting-growing temperature conditions as well.

## Figures and Tables

**Table 1:** Results of multivariate ANOVA: *F*-criteria and significance of fixed effects of rooting and growing conditions, clone, and their interaction during vegetative propagation in Phytotron on total phenolic compounds of *Populus* hybrids in clonal field trial. * Level of significance (*p*) of effects is denoted by *** *p* < 0.001; . = nonsignificant.

Effect	Num. *DF*	Den. *DF*	*F* Value	Prob *F*	p *
Growing conditions in Phytotron	1	114	241.49	<0.0001	***
Rooting conditions in Phytotron	2	114	2.35	0.1003	.
Interaction rooting × growing conditions	2	114	88.09	<0.0001	***
Interaction rooting conditions × clone	18	114	194.87	<0.0001	***
Interaction growing conditions × clone	9	114	93.43	<0.0001	***
Interaction rooting × growing conditions × clone	15	114	125.53	<0.0001	***

**Table 2:** Results of ANOVA (model 2): *F*-criteria and significance of fixed effects (treatments, hybrids, and their interaction) on different traits of *Populus* hybrids in clonal field trial. * Level of significance (*p*) of effects is denoted by: * 0.01 < *p* < 0.05; ** 0.001 < *p* < 0.01; *** *p* < 0.001; . = nonsignificant.

Effect	Num. *DF*	Den. *DF*	*F* Value	Prob *F*	p *
Height 2021					
Rooting–growing conditions in Phytotron	5	583	0.74	0.5941	.
Hybrid	4	583	0.62	0.6458	.
Interaction hybrid × rooting–growing conditions	20	583	2.23	0.0017	**
Height increment 2020					
Rooting–growing conditions in Phytotron	5	546	9.2	<0.0001	***
Hybrid	4	546	0.92	0.4537	.
Interaction hybrid × rooting–growing conditions	20	546	2.08	0.0041	**
Height increment 2021					
Rooting–growing conditions in Phytotron	5	550	1.56	0.1702	.
Hybrid	4	550	2.79	0.0257	*
Interaction hybrid × rooting–growing conditions	20	550	1.16	0.2843	.
Diameter 2021					
Rooting–growing conditions in Phytotron	5	583	1.21	0.3042	.
Hybrid	4	583	2.41	0.0483	*
Interaction hybrid × rooting–growing conditions	20	583	2.41	0.0006	***
Total phenolic compounds					
Rooting–growing conditions in Phytotron	2	141	0.52	0.5968	.
Hybrid	4	141	19.01	<0.0001	***
Interaction hybrid × rooting–growing conditions	8	141	6.29	<0.0001	***

**Table 3:** Stability characteristics of different poplar hybrids for total phenolic compounds and tree height in 2021.

Poplar Hybrid *	Hybrid Lsmean Deviation at Different Conditions						Phenotypic Plasticity															Wricke Ecovalence	Shukla Stability Variance		
	CR + CG *	CR + WG	WR + CG	WR + WG	HR + CG	HR + WG	CR + CG/CR + WG	CR + CG/WR + CG	CR + CG/WR + WG	CR + CG/HR + CG	CR + CG/HR + WG	CR + WG/WR + CG	CR + WG/WR + CG	CR + WG/ WR + WG	CR + WG/HR + CG	WR + CG/ WR + WG	WR + CG/HR + CG	WR + CG/HR + WG	WR + WG/HR + CG	WR + WG/ HR + WG	HR + CG/HR + WG		Variance	*F*	*p*
Total phenolic compounds																									
B × T	−6.0	−1.3	−3.7	−2.5	−2.7	−1.6	−4.7	−2.3	−3.5	−3.3	−4.4	2.4	1.2	1.4	1.4	−1.2	−1.0	−2.1	0.2	−0.9	−1.1	14.6	−2.5	−3.0	.
D × N	−1.2	−2.8	−0.8	−2.3	−0.4	2.0	1.5	−0.4	1.1	−0.8	−3.2	−1.9	−0.4	−2.3	−2.3	1.5	−0.4	−2.8	−1.9	−4.3	−2.4	14.2	−2.6	−4.9	.
D × T	1.5	5.8	−2.3	−2.6	5.4	−2.1	−4.3	3.7	4.1	−3.9	3.6	8.1	8.4	0.4	0.4	0.3	−7.7	−0.1	−8.0	−0.5	7.5	76.2	18.1	21.6	0
M × T	−2.2	2.3	0.4	1.9	−2.7	−0.1	−4.5	−2.6	−4.1	0.6	−2.0	1.9	0.4	5.0	5.0	−1.5	3.1	0.5	4.6	2.0	−2.6	21.1	−0.3	−0.4	.
T × T	11.0	−5.3	12.8	13.6	1.5	−0.2	16.3	−1.8	−2.5	9.6	11.3	−18.1	−18.9	−6.8	−6.8	−0.8	11.3	13.0	12.1	13.8	1.7	314.8	97.6	63.7	0
Tree height in 2021																									
B × T	−0.4	0.5	−0.3	0.1	−0.1	−0.2	−0.9	−0.1	−0.5	−0.3	−0.2	0.8	0.4	0.6	0.6	−0.4	−0.2	−0.1	0.2	0.3	0.1	0.5	0.6	37.3	0
D × N	0.2	−0.1	0.2	−0.3	0.1	−0.3	0.4	0.0	0.5	0.2	0.5	−0.4	0.1	−0.2	−0.2	0.5	0.2	0.5	−0.3	0.0	0.3	0.3	0.4	20.6	0
D × T	0.2	0.1	0.3	−0.1	0.0	−0.1	0.1	−0.1	0.3	0.2	0.4	−0.2	0.2	0.1	0.1	0.4	0.3	0.4	−0.1	0.1	0.1	0.1	0.2	10.9	0.009
M × T	−0.3	−0.2	−0.1	0.0	0.1	0.1	−0.2	−0.2	−0.4	−0.5	−0.4	0.0	−0.2	−0.3	−0.3	−0.2	−0.3	−0.2	−0.1	−0.1	0.0	0.2	0.3	12.4	0.001
T × T	−0.1	0.0	−0.2	0.2	−0.2	0.4	0.0	0.1	−0.2	0.1	−0.5	0.2	−0.2	0.1	0.1	−0.4	0.0	−0.6	0.3	−0.2	−0.5	0.3	0.3	18.8	0

* Rooting–growing condition abbreviations: cool rooting and cool growing conditions (CR + CG), cool rooting and warm growing conditions (CR + WG), warm rooting and cool growing conditions (WR + CG), warm rooting and warm growing conditions (WR + WG), hot rooting and cool growing conditions (HR + CG), and hot rooting and warm growing conditions (HR + WG). Hybrid-type abbreviations: B × T—*P. balsamifera* × *P. trichocarpa*, D × N—*P. deltoides* × *P. nigra*, D × T—*P. deltoides* × *P. trichocarpa*, M × T—*P. maximowiczii* × *P. trichocarpa*, T × T—*P. trichocarpa* × *P. trichocarpa*.

**Table 4:** Clonal variance components of different traits of *Populus* hybrids in clonal field trial affected by different rooting–growing treatments: trait means and clonal variance component. * Level of significance (*p*) of effects is denoted by: * 0.01 < *p* < 0.05.

Trait	Treatment *	Trait Mean ± se	Clonal Variance Component, ± se	p *	Clonal Variance Component, % ± se
Total phenolic compounds	CR + CG	18.48 ± 0.89	22.29 ± 11.32	*	95.33 ± 48.44
	CR + WG	16.29 ± 0.71	15.51 ± 7.46	*	94.04 ± 45.27
	WR + CG	18.96 ± 0.77	17.44 ± 8.76	*	98.63 ± 49.54
	WR + WG	15.44 ± 1.39	62.76 ± 29.63	*	99.55 ± 46.99
	HR + CG	17.19 ± 0.76	18.80 ± 8.88	*	99.38 ± 46.94
	HR + WG	16.38 ± 1.13	36.91 ± 18.52	*	98.93 ± 49.64

* Rooting–growing condition abbreviations: cool rooting and cool growing conditions (CR + CG), cool rooting and warm growing conditions (CR + WG), warm rooting and cool growing conditions (WR + CG), warm rooting and warm growing conditions (WR + WG), hot rooting and cool growing conditions (HR + CG), and hot rooting and warm growing conditions (HR + WG).

**Table 5:** Genetic parameters of different traits in 2021 of *Populus* hybrids in field trial affected by different rooting–growing treatments: coefficient of genotypic variation (*CV_G_*), coefficient of individual heritability (*H_i_*^2^), clonal mean heritability (*H_m_*^2^), and coefficient of phenotypic variation (*CV_F_*).

Trait	Treatment *	*CV_G_, %*	*H_i_*^2^ ± se	*H_m_*^2^ ± se	*CV_F_, %*
Height	CR + CG	15.1	0.11 ± 0.09	0.38 ± 0.11	45.35
	CR + WG	8.2	0.04 ± 0.07	0.20 ± 0.09	41.01
	WR + CG	6.6	0.02 ± 0.07	0.10 ± 0.09	47.01
	WR + WG	21.3	0.22 ± 0.10	0.61 ± 0.09	44.17
	HR + CG	15.8	0.11 + 0.12	0.32 ± 0.13	47.14
	HR + WG	19.6	0.11 ± 0.14	0.29 ± 0.15	59.95
Height increment	CR + CG	46.8	0.01 ± 0.08	0.03 ± 0.08	566.77
	CR + WG	212.5	0.09 ± 0.08	0.37 ± 0.00	691.42
	WR + CG	0.0	0.00 ± 0.07	0.00 ± 0.07	305.95
	WR + WG	81.1	0.17 ± 0.10	0.50 ± 0.07	195.98
	HR + CG	227.3	0.05 ± 0.11	0.17 ± 0.13	974.54
	HR + WG	107.9	0.19 ± 0.15	0.43 ± 0.14	245.45
Diameter	CR + CG	17.6	0.15 ± 0.10	0.46 ± 0.11	45.67
	CR + WG	13.2	0.09 ± 0.07	0.37 ± 0.10	45.26
	WR + CG	5.1	0.01 ± 0.07	0.05 ± 0.08	49.04
	WR + WG	33.0	0.42 ± 0.11	0.80 ± 0.06	48.9
	HR + CG	17.0	0.14 ± 0.12	0.36 ± 0.13	46.17
	HR + WG	21.7	0.16 ± 0.14	0.40 ± 0.14	53.02
Total phenolic compounds	CR + CG	25.5	0.95 ± 0.03	0.98 ± 0.01	25.19
	CR + WG	24.1	0.94 ± 0.03	0.98 ± 0.01	24.1
	WR + CG	22	0.99 ± 0.01	0.99 ± 0.00	21.32
	WR + WG	51.3	1.00 ± 0.00	1.00 ± 0.00	49.62
	HR + CG	25.2	0.99 ± 0.00	1.00 ± 0.00	24.42
	HR + WG	37	0.99 ± 0.01	1.00 ± 0.00	35.82

* Rooting–growing condition abbreviations: cool rooting and cool growing conditions (CR + CG), cool rooting and warm growing conditions (CR + WG), warm rooting and cool growing conditions (WR + CG), warm rooting and warm growing conditions (WR + WG), hot rooting and cool growing conditions (HR + CG), and hot rooting and warm growing conditions (HR + WG).

**Table 6:** Code list of hybrid poplar clones by crossing types and combination of crossed poplar species (only underlined clones were used in the total phenolic compounds study).

Crossing Type	Hybrid Abbreviation	Crossing Combination	Clone Number or Cultivar Name Abbreviation
Inter-specific	D × N	*P. deltoides × P. nigra*	Gr-Comp, Gr-Xe-3, Nyd-Elle, UK-AgatF, UK-Robus, UK-Spitk
	D × T	*P. deltoides × P. trichocarpa*	Isl-15, UK-Boela, UK-Donk
	M × T	*P. maximowiczii* × *P. trichocarpa*	SvSFPo2, SvSFPo6, SvSFPo7, UK-Andro
	B × T	*P. balsamifera* × *P. trichocarpa*	SvSFPo1, SvSFPo4, SvSFPo13
Intra-specific	T × T	*P. trichocarpa* × *P. trichocarpa*	SvSFPo14, SvSFPo15, UK-FrPau, SvSFPo9

## Data Availability

Not applicable.
